# GPCR-PEnDB: a database of protein sequences and derived features to facilitate prediction and classification of G protein-coupled receptors

**DOI:** 10.1093/database/baaa087

**Published:** 2020-11-20

**Authors:** Khodeza Begum, Jonathon E Mohl, Fredrick Ayivor, Eder E Perez, Ming-Ying Leung

**Affiliations:** Computational Science Program, The University of Texas at El Paso, 500 West University Avenue, El Paso, Texas 79968, USA; Border Biomedical Research Center, The University of Texas at El Paso, 500 West University Avenue, El Paso, Texas 79968, USA; Border Biomedical Research Center, The University of Texas at El Paso, 500 West University Avenue, El Paso, Texas 79968, USA; Bioinformatics Program, The University of Texas at El Paso, 500 West University Avenue, El Paso, Texas 79968, USA and; Department of Mathematical Sciences, The University of Texas at El Paso, 500 West University Avenue, El Paso, Texas 79968, USA; Computational Science Program, The University of Texas at El Paso, 500 West University Avenue, El Paso, Texas 79968, USA; Department of Mathematical Sciences, The University of Texas at El Paso, 500 West University Avenue, El Paso, Texas 79968, USA; Computational Science Program, The University of Texas at El Paso, 500 West University Avenue, El Paso, Texas 79968, USA; Border Biomedical Research Center, The University of Texas at El Paso, 500 West University Avenue, El Paso, Texas 79968, USA; Bioinformatics Program, The University of Texas at El Paso, 500 West University Avenue, El Paso, Texas 79968, USA and; Department of Mathematical Sciences, The University of Texas at El Paso, 500 West University Avenue, El Paso, Texas 79968, USA

## Abstract

G protein-coupled receptors (GPCRs) constitute the largest group of membrane receptor proteins in eukaryotes. Due to their significant roles in various physiological processes such as vision, smell and inflammation, GPCRs are the targets of many prescription drugs. However, the functional and sequence diversity of GPCRs has kept their prediction and classification based on amino acid sequence data as a challenging bioinformatics problem. There are existing computational approaches, mainly using machine learning and statistical methods, to predict and classify GPCRs based on amino acid sequence and sequence derived features. In this paper, we describe a searchable MySQL database, named GPCR-PEnDB (GPCR Prediction Ensemble Database), of confirmed GPCRs and non-GPCRs. It was constructed with the goal of allowing users to conveniently access useful information of GPCRs in a wide range of organisms and to compile reliable training and testing datasets for different combinations of computational tools. This database currently contains 3129 confirmed GPCR and 3575 non-GPCR sequences collected from the UniProtKB/Swiss-Prot protein database, encompassing over 1200 species. The non-GPCR entries include transmembrane proteins for evaluating various prediction programs’ abilities to distinguish GPCRs from other transmembrane proteins. Each protein is linked to information about its source organism, classification, sequence lengths and composition, and other derived sequence features. We present examples of using this database along with its graphical user interface, to query for GPCRs with specific sequence properties and to compare the accuracies of five tools for GPCR prediction. This initial version of GPCR-PEnDB will provide a framework for future extensions to include additional sequence and feature data to facilitate the design and assessment of software tools and experimental studies to help understand the functional roles of GPCRs.

**Database URL**: gpcr.utep.edu/database

## Introduction

G protein-coupled receptors (GPCRs) are a vast and diverse group of transmembrane receptor proteins in humans. GPCRs are involved in a wide range of physiological processes including vision, taste, smell and pain (1) and are implicated in many different diseases such as cancer (2), infection (3) and inflammation (4). Because of their critical roles in intracellular signaling and biomedical relevance, GPCRs are considered one of the most useful class of therapeutic targets (5). Indeed, it has been estimated that about 34% of FDA-approved drugs in the USA target GPCRs (6). Identification of GPCRs and understanding their molecular mechanisms have been the subject of many research studies (see, for example, the reviews articles (7, 8) and references therein).

**Figure 1.**
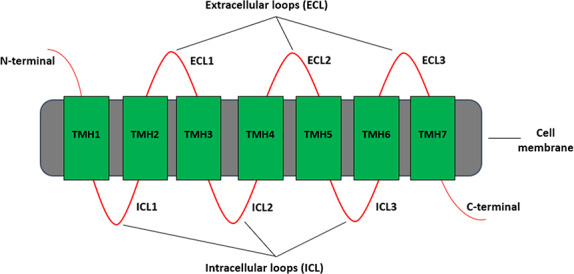
Different regions of a typical GPCR molecule. GPCR consists of a single polypeptide chain of amino acids folded into seven transmembrane helices (TMH1–7) between an extracellular N-terminal and an intracellular C-terminal. The seven transmembrane helices are connected by three extracellular loops (ECL1–3) and three intracellular loops (ICL1–3).

Each GPCR protein has a characteristic structure consisting of an extracellular N-terminal, an intracellular C-terminal, and between them seven hydrophobic transmembrane helices that are linked through three intracellular and three extracellular loops as shown in Figure 1. Based on this characteristic structure, many different bioinformatics software tools have been developed for predicting GPCRs and then classifying them hierarchically to gain insights into its possible biological functions. The sequences in GPCRdb (9), for example, are classified into families, subfamilies, sub-subfamilies and subtypes.

GPCRs are commonly grouped into families according to the International Union of Basic and Clinical Pharmacology (IUPHAR) (10) and Glutamate, Rhodopsin, Adhesion, Frizzled, Secretin (GRAFS) (11) systems. While IUPHAR applies to all GPCRs in general, the GRAFS system focuses more on vertebrate GPCRs. Table 1 displays the family names in the two systems and the correspondence between them. We extended the IUPHAR and GRAFS systems to include fungal pheromones, cyclic adenosine monophosphate (cAMP) receptors and the Taste 2 receptor families to account for GPCRs not covered by the standard classification systems. For simplicity, we will still refer to the extended systems by their original names in this paper.

**Table 1.** Number of GPCR sequences in the extended IUPHAR and GRAFS classification families

IUPHAR	GRAFS	No. of sequences
Class A	Rhodopsin-like	2493
Class B	Adhesion-like	91
	Secretin-like	113
Class C	Glutamate-like	112
Class D	Fungal pheromone^a^	13
Class E	cAMP receptor^a^	11
Class F	Frizzled	82
Class T2R^b^	Taste2 receptor^b^	211

a These invertebrate GPCR families are not in the original GRAFS system but are included here as descriptive labels corresponding to Classes D and E of the IUPHAR system.

^b^ This class is not in original IUPHAR or GRAFS classifications.

Aside from humans, GPCRs have been found in many different species including other mammals, insects, fungi, etc. As records of newly discovered GPCRs accumulate over the years, they have been collected in different databases as summarized in the study by Kowalsman and Niv (12). Some of these databases deal with proteins in general while others are specialized for GPCRs only. Among the specialized databases, GPCRdb (9) has served the scientific community for over 20 years, providing a comprehensive repository of GPCR sequence information spanning a large number of species. Currently, it contains over 15 000 proteins from more than 3500 species. Many prediction and classification programs take sequence data from GPCRdb as positive examples for their training and testing datasets. Another database is SeQuery (13), which allows users to visualize the GPCR families’ proteome or genome networks using a graph-based approach and analyze the relationship of a query sequence with the other GPCRs based on their structures and functions from published literature. The SeQuery database contains over 3100 reviewed GPCR sequences collected from UniProt (14).

Some GPCR prediction and classification tools rely on sequence similarities [e.g. BLAST (15)] or common sequence motif profiles [e.g. Pfam (16), PRINTS (17) and PROSITE (18)] in GPCRs, while others use machine learning or statistical classification algorithms (e.g. support vector machines, *K* nearest neighbors and decision trees). An informative compendium on the different computational approaches can be found in the study by Suwa (19). A web-based GPCR prediction and classification tool, called GPCR Prediction Ensemble (GPCR-PEn, accessible at gpcr.utep.edu), has been developed to let users select combinations of existing bioinformatics tools to perform GPCR prediction and classification on their own sequence data from different source organisms for different research objectives. For example, potential GPCRs were predicted from transcriptome data for the cattle ticks *Rhipicephalus microplus* and *Rhipicephalus australis* with the aim to facilitate development of new technologies for better control of these agricultural pests (20, 21). To estimate the collective performance of different combinations of prediction tools, it is necessary to have a unified and integrated dataset that satisfies the following basic requirements:

The dataset should contain both positive and negative examples of GPCRs.
There should be proteins from diverse taxonomic classes in the dataset.
Positive examples should comprise confirmed GPCRs supported by experimental evidence or curator verification.
Negative examples should span a large variety of proteins, including non-GPCR transmembrane proteins, with different structures and functions.

With the above requirements in mind, we have developed GPCRPEn-DB as a searchable database with confirmed positive examples of GPCRs and a variety of negative examples including non-GPCR transmembrane proteins. This paper describes the content, design and construction of the database along with its web-based user interface and demonstrates its application in assessing the accuracies for several GPCR prediction tools.

## Materials and methods

### Data collection

We retrieved proteins from the UniProt (Universal Protein Resource) database (14) at www.uniprot.org that provided protein sequence data and annotations. In particular, we used the data in the Swiss-Prot section of UniProtKB protein knowledgebase as they are better curated with supporting experimental evidence.

UniProt’s advanced search option was used to conduct a ‘Family and Domains’ search with the ‘protein family’ function. The search terms were ‘G protein-coupled receptor *n* family’ with *n* = 1,…,5. These searches retrieved all the GPCR sequences in the IUPHAR Classes A–E. The Class F and Taste 2 sequences were searched using ‘G protein-coupled receptor fz smo family’ and ‘G protein-coupled receptor T2R family,’ respectively. In each search, we included the ‘Reviewed Yes’ filter to select only those proteins that have been reviewed and confirmed to be GPCRs. Each family was downloaded and merged into one FASTA formatted file. The header line for each sequence gives the GRAFS then IUPHAR family classifications, along with the UniProt ID and entry name. For example, the protein sequence with header line

$$\begin{equation*}{\rm{ \gt Secretin\hbox{-}like}}\; \left|\; {{\rm{ Class\; B}}} \; \right|\; {\rm{ P34998\; |\; crfr1\_human}}\end{equation*}$$

belongs to the Secretin-like family by GRAFS classification, which corresponds to Class B in the IUPHAR nomenclature. The third section gives its UniProt ID, and the entry name in the fourth section says it is a human GPCR called crfr1. Later, we matched the IDs of our dataset with GPCRdb and downloaded the lower-level classification (subfamily, sub-subfamily, subtype) names for the sequences. As our GPCR collection contains sequences that are not currently in GPCRdb [e.g. the Rhodopsin-like (Class A) GPCRs such as the odorant/olfactory, and opsin receptors], only around 70% of our GPCRs were classified to are collected lower levels using GPCRdb.

The negative examples in our database were obtained by downloading all sequences from Swiss-Prot, which were not in GPCR families 1–5 or fz/smo using UniProt’s advanced search functions with the ‘protein families’ option and the Boolean argument ‘NOT’ is used to get the non-GPCRs. This collection of proteins was much larger than our GPCR dataset. To make it more comparable in size to our GPCR set, a random sample of 3000 sequences was taken from the collection. We then used the version of the CD-HIT program provided by UniProt (14) to cluster sequences with ≥50% sequence identity and only one representative was collected from each cluster.

To enhance our negative dataset, a collection of transmembrane non-GPCRs, we searched specifically for the ‘transmembrane’ proteins that are not classified as GPCRs. At first, the search is done using the Boolean argument ‘NOT G protein-coupled receptor family’ to avoid GPCR families. Using CD-HIT with a threshold of ≥50% ensures that the sequences obtained are sufficiently diverse while removing homologous sequences. Then using the ‘transmembrane’ property provided by Uniprot, only the proteins that have one or more transmembrane helices are selected. The sequences were again compiled into a FASTA file as described for the positive examples above. However, the header line contains the label ‘Negative’ instead of the GPCR family classification.

### Database implementation

GPCR-PEnDB is a relational database that contains information about each protein starting from general overview (e.g. name, id and gene), then different levels of classification, source organisms and protein features (e.g. amino acid and dipeptide percentages). To easily access information for both the positive and negative datasets, we have created seven tables, namely, Protein, Organism, AA_Dipeptide (Amino acid and dipeptide), TMHMM_Length (Transmembrane hidden Markov model length), IUPHAR, GRAFS and LL_classification (lower-level classification). The entity relationship diagram is shown in Supplemental Figure S1.

The Protein table (Primary key: Protein_ID) contains the sequence ids, protein names, entry names, alternative names, sequence lengths (in terms of number of amino acid residues), the indicator distinguishing GPCRs from non-GPCRs and the available PDB IDs of the GPCRs. In this table, IDs have been assigned to the protein sequences based on the GRAFS and IUPHAR system along with the IDs assigned for the organism types. These allow the proteins to connect with the GRAFS, IUPHAR and Organism tables respectively using the foreign keys defined in the table as GRAFS_ID, IUPHAR_ID, Organism_ID.

In the Organism table (Primary key: Organism_ID), all the entities have their scientific names and common names along with an identification number. For bacteria and viruses, serotype and strain information are also included. An additional column named ‘Frequency’ has the counts of the sequences available in the dataset for each type of organism. With this structure, user can construct datasets that focus on a set of specified organisms.

The GRAFS and the IUPHAR tables (Primary keys: GRAFS_ID, IUPHAR_ID) have the same structure with two columns. The first column contains the IDs and the second column has the family names of the classification system as shown in Table 1.

The LL-Class table (Primary key: Protein_ID) contains three fields to keep the lower level classification information of subfamily, sub-subfamily and subtype for each GPCR.

The AA_Dipeptide table (Primary key: Protein_ID) contains amino acid and dipeptide percentages. It has 423 columns, with the first one containing the protein name. The next 20 columns give the percentages of the common types of amino acids (represented as A, C, D,…,W, Y) and one more column for all other unidentified amino acids found in the sequences. These are followed by the percentages of the 400 dipeptides (AA, AC, AD, …., YW, YY) plus one column for all unidentified dipeptides.

GPCR structural features that include the lengths of the transmembrane helices, N- and C-terminals, as well as the inside and outside loops are important characteristics for prediction and classification. If the 3D structure of a GPCR is available, such information can be obtained from its record deposited in the Protein Data Bank (PDB). Unfortunately, relatively few 3D structures for GPCRs have been established to date. Our recent search through PDB has found only 546 3D structures related to 108 distinct GPCRs, corresponding to less than 4% of our GPCR collection. We have therefore decided to use the hidden Markov model based transmembrane helix prediction tool TMHMM2.0 (22) to estimate of the lengths of the structural regions for the GPCR dataset and generated the TMHMM_Length table (Primary key: Protein_ID). This table contains the predicted lengths of the N- and C-terminals, seven transmembrane helices, three inside and three outside loops for the GPCRs whenever the estimation is possible.

GPCR-PEnDB was implemented on a Dell PowerEdge R430 rack server that uses dual Intel Xeon E5-2620 processors and two 16-GB DIMM memory modules. The server utilizes the CentOS 7 operating system, a Red Hat Enterprise Linux derivative. The database was built with MySQL Version 14.14 Distribution 5.6.37, for Linux (x86_64) using EditLine wrapper.

### Web server interface

**Figure 5.**
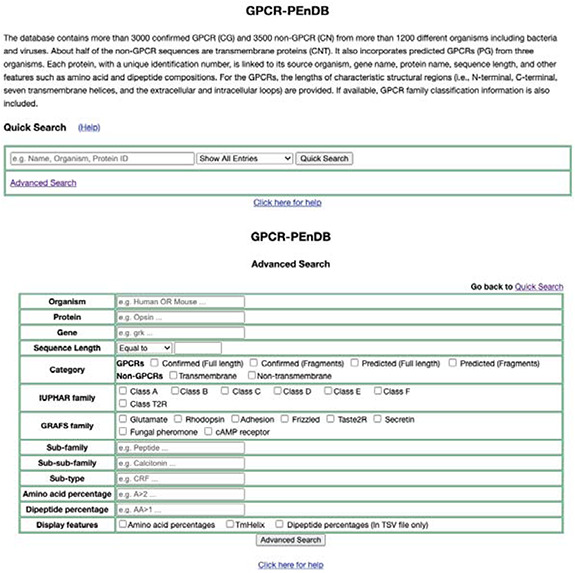
Web interface of GPCR-PEnDB, showing both Quick Search (top) and Advanced Search options (bottom).

A web interface for GPCR-PEnDB (Figure 5), implemented in the web.py framework (0.37 version), has been made publicly accessible at gpcr.utep.edu/database. This web server allows users who are not familiar with MySQL to generate queries easily by specifying different input search parameters. Two search options are available, quick and advanced. The quick search allows users to specify only one conditional clause (MySQL clause name: WHERE) from only a single table. The output will display information from all the tables for those proteins satisfying the search criterion. In the advanced search, multiple conditional clauses can be specified by the user to generate the query.

We have used Python scripts to transform the inputs specified by the user into an SQL query to gather the results from the database. These results are presented in the ‘Results Table’ page of the webserver in a tabular format. The saved outputs are used twice, first for writing the results in a TSV file and then for assembling the protein sequences in a FASTA file. Links are given to download both files. Clicking on the FASTA file link allows the user to apply the CD-HIT tool (23) to select a representative sequence from highly similar sequence clusters before downloading. This would ensure that the user is able to capture the desired diversity of the results while reducing the number of sequences downloaded.

### GPCR prediction tools assessment

We conducted a study on several available GPCR prediction tools to assess their performance using our confirmed positive and negative examples in GPCR-PEnDB. We downloaded and implemented the programs Pfam (16), GPCR-Pred (24) and GPCR-Tm (20, 21) and run them locally for this assessment and also evaluated PCA-GPCR (25) and SVMProt (26) via their public web servers. The following statistical measures were calculated:

$$\begin{eqnarray*}&&{\rm{Sensitivity}} = {\ }{{{\rm{True\; positives}}} \over {{\rm{True\; positives}} + {\rm{False\; negatives}}}}\\
&&{\rm{Specificity}} = {\ }{{{\rm{True\; negatives}}} \over {{\rm{True\; negatives}} + {\rm{False\; positives}}}}\\
&&{\rm{Positive\; Predictive\; Value }}\left( {{\rm{PPV}}} \right)\notag\\
&&\sim\sim\sim\sim\sim\sim\sim\sim\sim\sim\sim\sim\sim\sim\sim\sim\sim\sim\sim\sim = {\ }{{{\rm{True\; positives}}} \over {{\rm{True\; positives}} + {\rm{False\; positives}}}}\\
&&{\rm{Negative\; Predictive\; Value }}\left( {{\rm{NPV}}} \right)\notag\\
&&\sim\sim\sim\sim\sim\sim\sim\sim\sim\sim\sim\sim\sim\sim\sim\sim\sim\sim\sim\sim = {\ }{{{\rm{True\; negatives}}} \over {{\rm{True\; negatives}} + {\rm{False\; negatives}}}}\\
&&{\rm{Accuracy}}= {\ }{{{\rm{True\; positives}} + {\rm{True\; negatives}}} \over {{\rm{Total\; test\; sequences}}}}\end{eqnarray*}$$

In addition, we used all the transmembrane non-GPCR proteins to assess the transmembrane false positive rate (TmFPR) as given by

$$\begin{align*}&{\rm{TmFPR}}=\notag\\
& {\ }{{{\rm{ Transmembrane\; non\hbox{-}GPCRs\; falsely\; predicted\; as\; GPCRs}}} \over {{\rm{Total\; transmembrane\; non\hbox{-}GPCRs}}}}\end{align*}$$

A low TmFPR would indicate a good capability of the prediction tool to distinguish non-GPCR transmembrane proteins from GPCRs.

## Results and discussion

**Figure 2.**
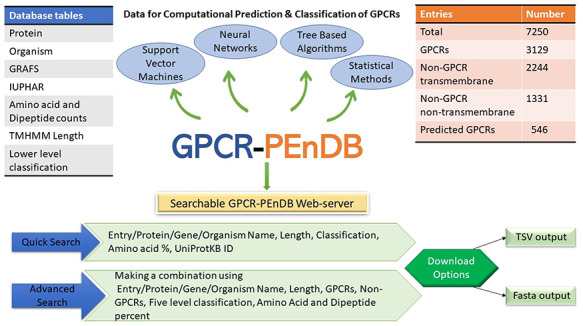
G protein-coupled receptor Prediction Ensemble Database (GPCR-PEnDB) overview showing the tables in the database, number of sequence entries, available web-server search options, and different types of algorithms for GPCR prediction and classification.

In this section, we describe the resulting database, give examples of different queries and demonstrate how the collected data can be used to assess the performance of different GPCR prediction tools. Figure 2 gives an overview of GPCR-PEnDB.

### Collected datasets of GPCRs and non-GPCRs

**Figure 3.**
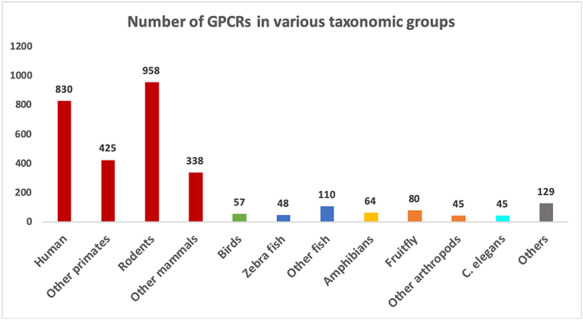
Number of sequences in different groups of organisms in the GPCR datasets. Groups with more than 40 sequences are shown as separate bars. The remaining ones are grouped as “Others”.

The collected data resulted in two FASTA files containing 3129 confirmed GPCRs and 3575 non-GPCRs. Table 1 shows the numbers of GPCR sequences grouped by the GRAFS and IUPHAR families. As expected, the vast majority of GPCRs belong to the rhodopsin-like family or Class A. Figure 3 shows the number of proteins available in the GPCR datasets grouped by major taxonomic classes. In total, there are 1290 distinct organism IDs, of which 289 are associated with GPCRs. It can be seen from Figure 3 that the GPCR collection is highly dominated by mammalian sequences.

The number of positive examples in our dataset is small compared to the GPCR collection in the established databases like GPCRdb that contains over 15 000 GPCR sequences. The difference is mainly due to our requirement for all positive examples to be confirmed GPCRs, which would best serve the purpose of evaluating different GPCR prediction and classification algorithms. On the other hand, our GPCR collection contains 1100 proteins that are not in the current GPCRdb. These are mainly receptors from Class A including olfactory, vomeronasal, tyramine, octopamine and opsins. For Class B we have incorporated methuselah and latrophilin types of proteins, and for Class C our database has some additional groups of metabotropic glutamate receptors not available in GPCRdb. Furthermore, GPCR-PEnDB also contains Classes D and E receptors, which are totally absent from GPCRdb, as well as some additional receptors from Class F. A comparison list of proteins in GPCRdb and GPCR-PEnDB is provided in Supplemental file S2.

The availability of negative examples is a unique feature of GPCR-PEnDB. Over 60% of these negative examples are non-GPCR transmembrane proteins. As GPCRs have seven transmembrane helices, they may share certain similarities with other transmembrane non-GPCRs with different or even the same number of helices. To distinguish GPCRs from other transmembrane proteins, it is important to have a good number of such sequences in the negative examples. The negative dataset is intentionally included to facilitate the construction of test datasets to evaluate the capability of any GPCR prediction program to separate GPCRs from other transmembrane proteins.

The current version of GPCR-PEnDB has also included unconfirmed GPCRs for three arthropods, *Anopheles gambiae* (mosquito), *Drosophila melanogaster* (fruit fly) and *Rhipicephalus microplus* (cattle tick), which are labeled as ‘predicted GPCRs.’ The unreviewed sequences for mosquito and fruit fly were retrieved from UniProt and the predicted cattle tick sequences are obtained from the supplemental materials of the study by Guerrero *et al*. (20). Although these organisms are of importance in biomedical and agricultural research, there are relatively few confirmed GPCR sequences for arthropods in general, as can be seen in Figure 3. We have planned to extend our database to further incorporate predicted GPCR data for more organisms. Such a predicted GPCR collection can be especially useful for researchers studying non-mammalian organisms where confirmed GPCRs are scanty.

### The searchable GPCR-PEnDB database

**Figure 4.**
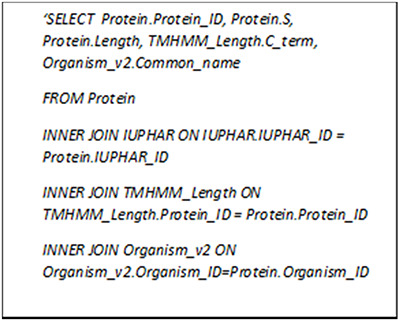
MySQL query asking for GPCRs in Class A with more than 10% serine and C-terminal longer than 300 amino acid residues.

We have gathered the general information (e.g. protein name, gene name and sequence) for each protein along with the common features like amino acid and dipeptide percentages. For the GPCRs, the family and lower-level classifications as well as the lengths of characteristic regions estimated by TMHMM 2.0 are also provided whenever possible. We can search GPCR-PEnDB by generating MySQL queries consisting of various clauses that not only involve joining multiple tables but grouping the results based on a numeric range. Figure 4 contains a query that search for all the GPCRs with ‘Class A’ IUPHAR classification, >10% serine in amino acid composition and >300 in C-terminal length. The search result, as shown in Table 2, also displays the UniProt protein ID, sequence length and the source organism.

**Table 2.** Output table of the query asking for GPCRs in Class A with more than 10% serine and C-terminal longer than 300 amino acid residues

Protein_ID	Serine(S) %	Length	C_term	Common name
Q9W534	10.91	670	305	Fruit fly
Q6NV75	10.18	609	311	Human
Q86SP6	12.31	731	367	Human
Q8K0Z9	10.30	631	333	Mouse
Q9DDD1	12.31	723	357	Chicken
Q924Y8	11.92	730	368	Rat

It should be noted here that we have encountered a couple of problems in obtaining complete information for some of the GPCR sequences. First, lower-level classifications are not available for our 1100 GPCRs that are not in GPCRdb. Although we have tried using some existing GPCR classification tools, their classification systems were not totally consistent with that used in GPCRdb. Second, when TMHMM 2.0 was used to estimate the lengths of characteristic regions, the program predicted the number of helices erroneously as six or eight rather than seven for 550 of the full-length GPCRs in our database. We also attempted to look into UniProt for the regional length information but there were still issues such as the exact length of a helix or N-terminal being missing or the reported lengths being inconsistent with the common structure of GPCRs. Due to these reasons current GPCR-PEnDB can only provide estimated regional lengths for those GPCRs that were predicted with seven transmembrane helices by TMHMM 2.0.

### Web interface for GPCR-PEnDB

The web interface (Figure 5) is designed to provide the flexibility for users to obtain information from GPCR-PEnDB without constructing MySQL queries. One can assemble different queries and narrow down the search to accumulate details about the entities of interest by entering or selecting parameters on the webpage. Each resulting protein ID is linked to the UniprotKB database for the user to find more detailed information about the protein. The user can also specify whether or not the display should include detailed information of amino acid percentages or the structural region lengths estimated by TMHMM. From the results page, users can compile and download the tabular results in TSV format and sequences in a FASTA format. The FASTA download can be done with or without using the clustering tool CD-HIT that provides nonredundant representative sequences as output. This allows user to keep the FASTA sequences separated from the sequence derived features so that other features can be generated and used if needed.

**Figure 6.**
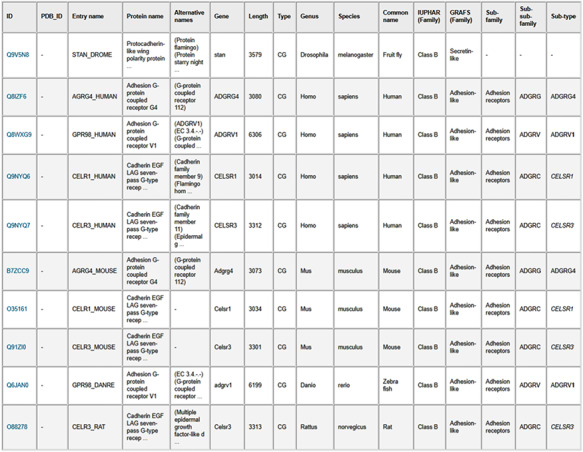
Results table from the search of GPCR sequences longer than 3000 amino acids using the web server. The table entries can be downloaded in CSV format by clicking on the “Result table” link, and the corresponding protein sequences can be downloaded in FASTA format by clicking on the “FASTA file” link.

As an example to illustrate using the advanced search option, we can look for all GPCR sequences with lengths greater than 3000 (see Figure 5). The search result in Figure 6 shows that there are 10 such confirmed GPCR sequences from different organisms such as human, fruit fly, mouse, zebra fish and rat. All these sequences belong to Class B (Secretin-like/Adhesion-like family). This tells us that GPCRs from other families do not exceed 3000 in length. The lower-level classification information is also shown for all but one of these GPCRssess the prediction accuracies of sevs. Furthermore, if we choose the option to display the available TMHMM predicted regional lengths, we can see that the N-terminals (length 2470–5907) are much longer than the C-terminals (length 92–315) in these long Class B GPCRs.

### Assessment of GPCR prediction tools

Using our compiled data in GPCR-PEnDB, we conducted a study to assess the prediction accuracies of several GPCR prediction and classification tools. Our investigation was motivated by a preliminary smaller scale exercise where we applied various GPCR prediction tools on 10 transmembrane non-GPCR proteins and observed that a large portion of them were erroneously predicted as GPCRs. The programs assessed include the hidden Markov model–based Pfam (16) and GPCR-Tm (20, 21), the support vector machine–based GPCRpred (24) and SVM-Prot (26), and PCA-GPCR (25) that combines principal component analysis with an intimate sorting algorithm. All these programs are either accessible through a public website or have source code available that can be downloaded and implemented on a local machine. Among them, GPCRPred, GPCR-Tm and PCA-GPCR were developed specifically for GPCR prediction but Pfam and SVM-Prot are general tools for functional classification of proteins.

The performances of these tools (see Table 3), with overall accuracies ranging from around 73–97%, are considered satisfactory to excellent. However, we have also observed that the false positive rates among transmembrane non-GPCRs are very high for all the three GPCR-specific prediction tools. In contrast, the general-purpose Pfam and SVM-Prot performed much better. This may be attributed to the availability of a much larger variety of non-GPCR proteins in the training data for Pfam and SVM-Prot. However, because these programs were not designed for GPCR prediction, their outputs have to go through several additional post-processing steps before one can decide whether an input protein sequence is a GPCR or not. So, reducing the high TmFPR in the current GPCR prediction tools can be a desirable improvement.

**Table 3.** Assessment on available web-servers (all numbers reported are percentages)

	Pfam	GPCRPred	GPCR-Tm	PCA-GPCR	SVM-Prot
**Accuracy**	90.95	86.32	88.76	72.90	96.57
**Sensitivity**	80.02	97.98	95.82	99.62	96.57
**Specificity**	99.72	76.95	83.09	51.60	96.56
**^a^** **PPV**	99.57	77.35	81.99	62.14	95.77
**^a^** **NPV**	86.13	97.93	96.11	99.42	97.22
**^a^** **TmFPR**	0.45	36.83	26.98	70.64	5.76

a PPV: positive predictive value, NPV: negative predictive value, TmFPR: false positive rate among transmembrane non-GPCRs.

It should be noted that some of the GPCR prediction programs can, in varying degrees, classify GPCRs into finer levels. For example, using the reviewed GPCR sequences from Uniprot and a clustering approach, SeQuery (13) can generate, for a given GPCR, its centrality relationships with other closely related protein sequences at three different levels (individual protein, subfamily and family). Nevertheless, the classification systems used in the various programs are not all the same and each has its individual restrictions. We have not yet come across one that can perform a full classification of general GPCR proteins reliably all the way down from the family to the subtype level. One possible reason could be due to the limited number of confirmed examples of in the Class D, E and F families. We expect that appropriate use of over- and under-sampling techniques (27, 28) should help circumvent this problem of data imbalance. The sequence entries in GPCR-PEn will conveniently provide data to facilitate such algorithm development work.

### Comparison of GPCR-PEnDB with other databases

In Table 4, we provide a comparison of the features and capabilities of GPCR-PEnDB with UniProt, GPCR-PEnDB and SeQuery databases, showing some unique features incorporated in our database that help provide analysis-ready datasets for users to test the performances of existing or newly developed algorithms The provision of diverse confirmed GPCR and non-GPCR examples and the capability of searching by both GRAFS and IUPHAR classifications are most notable characteristics of our database. Furthermore, as our purpose is to facilitate GPCR prediction and classification, GPCR-PEnDB also provides some additional search criteria to help user ensure the obtained datasets only contains the appropriate sequences. For example, a search criterion can be set to screen out sequences containing undetermined amino acid residues, which are not allowed by some algorithms (e.g. SVMProt). Other details are listed in Table 4.

**Table 4.** Comparison of GPCR-PEnDB with UniProt, GPCRdb, and SeQuery

	UniProt	GPCRdb	SeQuery	GPCR-PEnDB
**Overview**	Database of protein sequences and their biological information	Collection of data, diagrams and webtools for analyzing GPCR structures and phylogenetic relationships	Graphical visualization database to analyze genome/proteome networks	Database of confirmed GPCRs and non-GPCR examples to facilitate prediction and classification of GPCRs
**GPCR sequence collection**	Mixed reviewed/unreviewed GPCRs	Mixed reviewed and unreviewed GPCRs from UniProt, excluding olfactory receptors	Reviewed GPCRs collected from UniProt, GPCRdb and PDB.	Reviewed GPCRs from UniProt, unreviewed GPCRs from a few species
**Non-GPCR sequence collection**	Mixed reviewed/unreviewed non-GPCRs	Not available	Not available	Reviewed non-GPCRs, including Tm and non-Tm proteins
**GRAFS & IUPHAR classification**	Not available	Searchable, single sequence can be downloaded using numeric code	Not available	Searchable, sequences can be downloaded by classification
**Searchable labels for GPCR and non-GPCR**	Not available	Not available	Not available	Confirmed/predicted GPCRs; Tm/nonTm non-GPCRs; full GPCRs/fragments;
**Sequence selection by CDHIT**	Available	Not available	Not available	Available
**Sequence features**	GPCR regional lengths	Not available	Not available	Amino acid and dipeptide percentages; TMHMM2.0 estimates of GPCR regional lengths
**Sequences with nonstandard amino acids**	Not indicated	Not indicated	Not indicated	Indicated and separable from other sequences
**Sequence download options**	FASTA, TSV, RDF/XML, Excel, Text, GFF	JSON, API	Not available	FASTA, TSV
**Structural information**	Links to PDB	Available	Not available	Links to PDB
**Ligand information**	Available	Available	Not available	Not available
**Information display**	Text, tables and figures	Text, tables, and figures	Text and figures	Tables

## Conclusion and future work

We have set up the GPCR-PEnDB database along with a user-friendly web interface that would allow users to easily search for the sequence and related information of confirmed GPCR and non-GPCR proteins. It allows users, according to their own research interests, to compare and contrast sequence features among different groups of GPCRs, and to compile datasets for training, testing and evaluation of GPCR prediction and classification algorithms.

With this initial version, GPCR-PEnDB provides the necessary framework for growth and refinement as more information can be included and their display can be improved in future developments. Ongoing work to expand the sequences within the database includes extending the collection of predicted but not yet confirmed GPCRs, as well as incorporating 3D structural information, ligand-binding sites and available gene ontology information (GO-terms) that identifies the biological processes and molecular functions involved in order to help elucidate the functional roles of individual GPCRs.

## Supplementary Material

baaa087_Supp
