# Supplementary material for: GPCR-PEnDB: a database of protein sequences and derived features to facilitate prediction and classification of G protein-coupled receptors
Source: Database (Oxford). 2020 Nov 20;2020:baaa087. doi: 10.1093/database/baaa087 (PMC7678784; doi:10.1093/database/baaa087)
Supplement: baaa087_Supp [file baaa087_supp.zip › S2.docx]

| Class A | |
| --- | --- |
| Receptors that are not in GPCRdb but available in GPCR-PEnDB | |
| - Ultraviolet-sensitive opsin - Putative tyramine receptor - Putative vomeronasal receptor-like protein - Putative gustatory receptor clone - Putative G-protein coupled receptor - Putative gonadotropin-releasing hormone II receptor - Putative violet-sensitive opsin - Retinochrome - Tyramine/octopamine receptor - viral G-protein coupled receptor - Violet-sensitive opsin - Vomeronasal type-1 receptor - Tyramine receptor - Red-sensitive opsin- - Putative olfactory receptor - Putative neuropeptide Y receptor - Tachykinin-like peptides receptor | - Ocellar opsin - Octopamine receptor, 1,2, beta-1R,-2R,-3R, Oamb - Octopressin receptor - Odorant receptor 131 - Olfactory receptor - Protein trapped in endoderm-1 - Protein UL78 - Pyrokinin-1 receptor - QRFP-like peptide receptor - RPE-retinal G protein-coupled receptor - RYamide receptor - Sex peptide receptor - Trissin receptor - Vertebrate ancient opsin - Visual pigment-like receptor peropsin - Serpentine receptor |

**Receptors in GPCRdb and GPCR-PEnDB**

| Class B1 (Secretin) | | |
| --- | --- | --- |
| Both in GPCRdb and GPCR-PEnDB | In GPCR-PEnDB but are not in GPCRdb | In GPCRdb but are not in  GPCR-PEnDB |
| - Calcitonin gene-related peptide type 1 receptor - Calcitonin receptor - Corticotropin-releasing factor receptor 1,2 - Gastric inhibitory polypeptide receptor - Glucagon receptor - Glucagon-like peptide 1 receptor - Growth hormone-releasing hormone receptor - Parathyroid hormone 2 receptor - Parathyroid hormone/parathyroid hormone-related peptide receptor - Pituitary adenylate cyclase-activating polypeptide type I receptor - Secretin receptor - Vasoactive intestinal polypeptide receptor Vasoactive intestinal polypeptide receptor 2 | - Calcitonin receptor-like protein 1 - Diuretic hormone receptor (DH-R) - G-protein coupled receptor 157 - G-protein coupled receptor Mth, Mth2 (Protein methuselah) - G-protein coupled receptor seb-2 - Latrophilin Cirl - Latrophilin receptor-like protein A - Latrophilin-like protein 1,2 - PDF receptor (Pigment-dispersing factor receptor) (Protein groom-of-PDF) - Probable G-protein coupled receptor Mth-like 1-14 | - ADCYAP receptor type I |
|  |  | - Adenylate cyclase activating polypeptide 1 (Pituitary) receptor type I |
|  |  | - calcitonin gene-related peptide type 1 receptor |
|  |  | - Calcitonin receptor, isoforms, types, fragments, like-receptors |
|  |  | - Corticotropin releasing hormone receptor 1,2 |
|  |  | - Deleted |
|  |  | - G protein-coupled pituitary GHRH receptor (Growth hormone-releasing hormone receptor) |
|  |  | - Gastric inhibitory polypeptide receptor |
|  |  | - Glucagon like peptide 1,2 receptor - Glucagon receptor |
|  |  | - Parathyroid hormone 1,2 receptor |
|  |  | - parathyroid hormone/parathyroid hormone-related peptide receptor, isoforms |
|  |  | - Secretin receptor |
|  |  | - Uncharacterized protein (290) |
|  |  | - Vasoactive intestinal peptide receptor 1,2 - Vasoactive intestinal polypeptide receptor 1,2 |
|  |  | - VPAC1 receptor |

| Class B2 (Adhesion) | | | |
| --- | --- | --- | --- |
| Both in GPCRdb and GPCR-PEnDB | In GPCR-PEnDB but are not in GPCRdb | In GPCRdb but are not in  GPCR-PEnDB | |
| - Adhesion G protein-coupled receptor A1-A3 (G-protein coupled receptor 123-125) - B1-B3 - D1-D2 - E1-E4 - F1-F5 - G1-G7 - L1-L4 - V1 - G-type receptor 1-3 - (CD antigen CD97) - Putative adhesion G protein-coupled receptor E4P - Cadherin EGF LAG seven-pass G-type receptor 1-3 | - Adhesion G protein-coupled receptor A1 (G-protein coupled receptor 123) *Mouse* - B1 (Brain-specific angiogenesis inhibitor 1) *Rat, Mouse* - D1 (G-protein coupled receptor 133) *Mouse* - E2 (EGF-like module receptor 2) *Dog* - F2 (G-protein coupled receptor 111) *Rat, mouse* | - A1-A3 | - Other species except human, zebra fish, mouse, rat |
|  |  | - B1-B3 | - (Same as above) |
|  |  | - D1-D2 | - (Same as above) |
|  |  | - E1-E4 | - (Same as above) |
|  |  | - F1-F5 | - (Same as above) |
|  |  | - G1-G7 | - (Same as above) |
|  |  | - L1-L4 | - (Same as above) |
|  |  | - V1 | - (Same as above) |
|  |  | - Deleted | |
|  |  | - latrophilin-1,2,3, isoforms | |
|  |  | - probable G-protein coupled receptor 113, 125 | |
|  |  | - Protocadherin-like wing polarity protein stan | |
|  |  | - Unreviewed proteins | |

| Class C (Metabotropic Glutamate) | | |
| --- | --- | --- |
| Both in GPCRdb and GPCR-PEnDB | In GPCR-PEnDB but are not in GPCRdb | In GPCRdb but are not in GPCR-PEnDB |
| - Extracellular calcium-sensing receptor (CaSR) - Gamma-aminobutyric acid type B receptor subunit 1,2 - G-protein coupled receptor family C group 6 member A, B, C, D - Metabotropic glutamate receptor 1-8 - Probable G-protein coupled receptor 156, 158, 179 - Probable metabotropic glutamate receptor mgl-1 - Retinoic acid-induced protein 3 - Taste receptor type 1 member 1,2,3 (G-protein coupled receptor 70) | - Gamma-aminobutyric acid type B receptor subunit 1,2 (not for C elegans) - G-protein coupled receptor family C group 6 member A (don’t have from bovine goldfish and zebrafish) - (Odorant receptor 5.24), (Odorant receptor ZO6)   - *Bovine* - Probable G-protein coupled receptor CG31760 - Protein bride of sevenless - Vomeronasal type-2 receptor 1, 26, 116 - Metabotropic glutamate receptor (DmGluRA) - Metabotropic glutamate receptor-like protein A-R | - Calcium polyvalent cation receptor 1 (extracellular calcium-sensing receptor isoform X1) - Calcium sensing receptor - CASR isoform 1 (CASR isoform 2) (Calcium sensing receptor) - Deleted - Extracellular calcium sensing receptor - G protein-coupled receptor class C group 6 member A, isoform X1-X3 - Chimpanzee, - Horse, - Green anole, American chameleon, - Little brown bat, - Western lowland gorilla, - Dog, - Wild turkey, - Mallard, Anas boschas, Zebra fish - Gabbr2 protein, isoform 1 - Gamma-aminobutyric acid (GABA) B receptor, 2 - Glutamate metabotropic receptor 1-8, Glutamate receptor, metabotropic 1-8b, isoforms, - G-protein coupled receptor T1R3 - GRM proteins and isoforms (1-7), - LOC100127664 protein, - LOW QUALITY PROTEIN: metabotropic glutamate receptor 1, 6 - metabotropic glutamate receptor 1-8, isoforms, fragments - Taste 1 receptor member 1-3, types, isoforms - Uncharacterized protein (261) |

| Class F (Frizzled) | |
| --- | --- |
| Both in GPCRdb and GPCR-PEnDB | In GPCR-PEnDB but are not in GPCRdb |
| - Frizzled-1 (Fz-1) (cFz-1) - Frizzled-1 (Fz-1) (hFz1) (FzE1) - Frizzled-1 (Fz-1) (mFz1) - Frizzled-1 (Fz-1) (rFz1) - Frizzled-1 (Fz-1) (Xfz1) - Frizzled-10 (Fz-10) (CD antigen CD350) - Frizzled-10 (Fz-10) (cFz-10) - Frizzled-10 (Fz-10) (hFz10) (FzE7) (CD antigen CD350) - Frizzled-2 (Fz-2) (cFz-2) (Fragment) - Frizzled-2 (Fz-2) (hFz2) (FzE2) - Frizzled-2 (Fz-2) (mFz2) (Frizzled-10) (Fz-10) (mFz10) - Frizzled-2 (Fz-2) (rFz2) - Frizzled-2 (Fz-2) (Xfz2) - Frizzled-3 (Fz-3) (hFz3) - Frizzled-3 (Fz-3) (mFz3) - Frizzled-3 (Fz-3) (Xfz3) - Frizzled-4 (Fz-4) (cFz-4) - Frizzled-4 (Fz-4) (hFz4) (FzE4) (CD antigen CD344) - Frizzled-4 (Fz-4) (mFz4) (CD antigen CD344) - Frizzled-4 (Fz-4) (rFz4) (CD antigen CD344) - Frizzled-4 (Fz-4) (Xfz4) - Frizzled-5 (Fz-5) - Frizzled-5 (Fz-5) (hFz5) (FzE5) - Frizzled-5 (Fz-5) (mFz5) - Frizzled-5 (Fz-5) (Xfz5) - Frizzled-6 (Fz-6) - Frizzled-6 (Fz-6) - Frizzled-6 (Fz-6) (hFz6) - Frizzled-6 (Fz-6) (mFz6) - Frizzled-7 (Frz7) (Fz-7) - Frizzled-7 (Fz-7) (cFz-7) - Frizzled-7 (Fz-7) (hFz7) (FzE3) - Frizzled-7 (Fz-7) (mFz7) - Frizzled-8 (Fz-8) - Frizzled-8 (Fz-8) (hFz8) - Frizzled-8 (Fz-8) (mFz8) - Frizzled-8 (Fz-8) (Xfz8) - Frizzled-9 (Frizzled-like protein 9) (rFz9) - Frizzled-9 (Fz-9) (cFz-9) (Fragment) - Frizzled-9 (Fz-9) (hFz9) (FzE6) (CD antigen CD349) - Frizzled-9 (Fz-9) (mFz3) (mFz9) (CD antigen CD349) - Protein smoothened (SMOH) (Smooth) (dSMO) - Smoothened homolog (SMO) - Smoothened homolog (SMO) - Smoothened homolog (SMO) (Fragment) - Smoothened homolog (SMO) (Protein Gx) | - Frizzled (Frizzled-1) (dFz1) - Frizzled (Frizzled-1) (dFz1) - Frizzled and smoothened-like protein A - Frizzled and smoothened-like protein B - Frizzled and smoothened-like protein C - Frizzled and smoothened-like protein D - Frizzled and smoothened-like protein E - Frizzled and smoothened-like protein F - Frizzled and smoothened-like protein G - Frizzled and smoothened-like protein H - Frizzled and smoothened-like protein J (Cell number regulator protein A) - Protein mom-5 - Frizzled and smoothened-like protein K - Frizzled and smoothened-like protein L - Frizzled and smoothened-like protein M - Frizzled and smoothened-like protein N - Frizzled and smoothened-like protein O - Frizzled and smoothened-like protein P - Frizzled and smoothened-like protein Q - Frizzled/smoothened-like sans CRD protein A - Frizzled/smoothened-like sans CRD protein B - Frizzled/smoothened-like sans CRD protein C - Frizzled/smoothened-like sans CRD protein D - Frizzled/smoothened-like sans CRD protein E - Frizzled/smoothened-like sans CRD protein F - Frizzled/smoothened-like sans CRD protein G - Frizzled/smoothened-like sans CRD protein H - Frizzled/smoothened-like sans CRD protein J - Frizzled-10-A (Fz-10A) (Xfz10-A) - Frizzled-10-B (Fz-10B) (Xfz10-B) (Frizzled-9) (Fz-9) (Xfz9) - Frizzled-2 - Frizzled-2 (dFz2) - Frizzled-3 (dFz3) - Frizzled-3 (Fz-3) (cFz-3) (Fragment) - Frizzled-4 (dFz4) - Frizzled-6 (Fz-6) (cFz-6) (Fragment) - Frizzled-7-A (Fz-7-A) (Xfz7-A) - Frizzled-7-B (Fz-7-B) (Xfz7-B) - Frizzled-8 (Fz-8) (cFz-8) (Fragment) - Protein mom-5 |

| Taste 2 Receptors | |
| --- | --- |
| Both in GPCRdb and GPCR-PEnDB | In GPCR-PEnDB but are not in GPCRdb |
| - Taste receptor type 2 member 1, 3, 4, 5, 7, 8, 9, 10, 13, 14, 16, 19, 20, 30, 31, 38, 39, 40, 41, 42, 43, 45, 46, 50, 60, 70, 103, 105, 106, 107, 109, 110, 113, 114, 119, 123, 140, 143, | - Taste receptor type 2 member 40, 62, 64, 66, 102, 103, 104, 105, 106, 107, 109, 110, 113, 114, 116, 117, 120, 123, 124, 125, 129, 134, 135, 136, 143 |
