# Supplementary figures and images for: GPCR-PEnDB: a database of protein sequences and derived features to facilitate prediction and classification of G protein-coupled receptors
Source: Database (Oxford). 2020 Nov 20;2020:baaa087. doi: 10.1093/database/baaa087 (PMC7678784; doi:10.1093/database/baaa087)

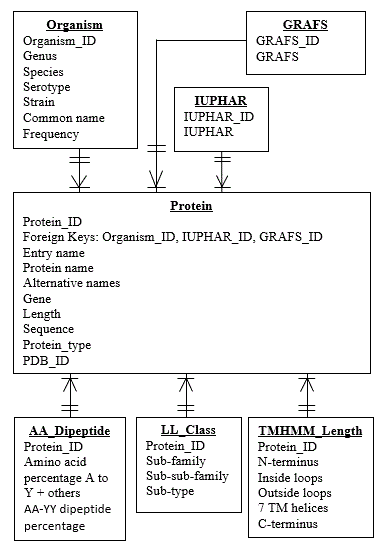

Supplement: baaa087_Supp [file baaa087_supp.zip › S1.GIF]
